# Transposon activation mutagenesis as a screening tool for identifying resistance to cancer therapeutics

**DOI:** 10.1186/1471-2407-13-93

**Published:** 2013-02-27

**Authors:** Li Chen, Lynda Stuart, Toshiro K Ohsumi, Shawn Burgess, Gaurav K Varshney, Anahita Dastur, Mark Borowsky, Cyril Benes, Adam Lacy-Hulbert, Emmett V Schmidt

**Affiliations:** 1 Center for Molecular Therapeutics, Center for Cancer Research, Massachusetts General Hospital, and Harvard Medical School, CNY 149-Rm7308, Thirteenth St, Charlestown, MA 02129, USA; 2 Program of Developmental Immunology, Massachusetts General Hospital, and Department of Pediatrics, Harvard Medical School, Boston, MA 02115, USA; 3 Department of Molecular Biology, Massachusetts General Hospital, and Department of Genetics, Harvard Medical School, Boston, MA 02115, USA; 4 Developmental Genomics Section, Genome Technology Branch, NHGRI, Bethesda, MD 20892, USA

**Keywords:** Transposon mutagenesis, Chemotherapy, Resistance, Gene activation

## Abstract

**Background:**

The development of resistance to chemotherapies represents a significant barrier to successful cancer treatment. Resistance mechanisms are complex, can involve diverse and often unexpected cellular processes, and can vary with both the underlying genetic lesion and the origin or type of tumor. For these reasons developing experimental strategies that could be used to understand, identify and predict mechanisms of resistance in different malignant cells would be a major advance.

**Methods:**

Here we describe a gain-of-function forward genetic approach for identifying mechanisms of resistance. This approach uses a modified piggyBac transposon to generate libraries of mutagenized cells, each containing transposon insertions that randomly activate nearby gene expression. Genes of interest are identified using next-gen high-throughput sequencing and barcode multiplexing is used to reduce experimental cost.

**Results:**

Using this approach we successfully identify genes involved in paclitaxel resistance in a variety of cancer cell lines, including the multidrug transporter *ABCB1,* a previously identified major paclitaxel resistance gene. Analysis of co-occurring transposons integration sites in single cell clone allows for the identification of genes that might act cooperatively to produce drug resistance a level of information not accessible using RNAi or ORF expression screening approaches.

**Conclusion:**

We have developed a powerful pipeline to systematically discover drug resistance in mammalian cells *in vitro*. This cost-effective approach can be readily applied to different cell lines, to identify canonical or context specific resistance mechanisms. Its ability to probe complex genetic context and non-coding genomic elements as well as cooperative resistance events makes it a good complement to RNAi or ORF expression based screens.

## Background

The development of resistance to cancer therapeutics represents a major hindrance to the successful pharmacological treatment and eradication of tumors in patients. Although some progress has been made in combining or augmenting treatments to counteract resistance, a major obstacle is our limited understanding of the mechanisms of resistance to current or novel therapeutics. Drug resistance can be mediated by further genetic and/or epigenetic changes in the tumor and, with the advent of high throughput sequencing, it is now feasible to systematically survey mutations in tumor genomes from patients following resistance development. However, the identification of the relevant ‘driver’ mutations, and other potential targets in resistance pathways, remains challenging.

A complementary approach is to identify resistance pathways experimentally using *in vitro* culture or animal model systems. Findings from such studies can then be used to inform analysis of patient samples and develop therapies to counteract resistance. Direct experimental identification of resistance genes has focused largely on reverse genetic and chemical biology approaches, including cDNA and RNAi library screens [1,2] or combined small molecule inhibitor and siRNA screens [3]. Such approaches can require expensive reagents and specialized platforms, and the need to consistently deliver siRNAs limits their applicability. Perhaps more importantly, as reverse genetic approaches, they are biased toward previously characterized genetic elements.

Forward genetic approaches using mobile genetic elements provide a powerful alternative method for gene discovery that can overcome many of the limitations of reverse genetic approaches. Mutagenesis with mobile genetic elements that insert into the genome offers a great scope for screening as these provide readily detected tags to identify insertion sites, and can potentially either activate or disrupt gene expression. Retroviruses have been used for insertional mutagenesis to identify oncogenes and study therapeutic resistance in tumors [4-6], however they preferentially insert in regions of open chromatin and high gene expression, leading to potential bias in results from genome-wide screens. Furthermore, the requirements for viral long terminal repeats (LTRs) and other structural restrictions limit the use of complex DNA constructs, limiting its applications to loss-of-function mutagenesis [7] and specialized haploid cell lines [8].

Transposons, another class of mobile genetic elements [9], have increasingly been utilized as genetic tools in mammals after the discovery and engineering of two transposons, Sleeping Beauty (SB) and piggyBac (PB) [10-13]. A major advantage of transposons is the simplicity of their integration machinery, which permits the incorporation of long DNA sequences, including functional genetic elements such as promoters, transcriptional stops and splicing sequences. This flexibility has allowed development of a variety of powerful mutagenesis schemes [14,15]. In their simplest application, transposons disrupt genes leading to loss of function, logically analogous to RNAi screens. With the incorporation of splice acceptors and reporter genes, transposons can also be used as an alternative to retroviral gene-traps [16,17]. Such gene disruption approaches are the basis for genome-wide insertion libraries in mouse embryonic stem cells [14,18]. Alternatively, inclusion of functional promoters within the transposon creates “activation tags” that cause expression of genes in which they land [19]. Activation tagging has been used in mouse somatic models to identify oncogenes [20,21]. This approach has great potential for gene discovery as it combines the strong phenotype of ‘gain-of-function’ approaches with the ability to probe the entire genome, including novel or uncharacterized genes and transcripts.

Here we report the development of transposon-based gene activation tagging for discovery of chemotherapeutic resistance genes. We constructed an activation PB transposon, generated mutagenesis libraries from several cancer cell lines, and characterized the mutations by sample barcoding and high-throughput sequencing. We validated this system by screening for genes involved in resistance to the microtubule targeting drug paclitaxel and identifying the multidrug resistance (MDR) gene *ABCB1* as the primary gene target. Through further analysis of individual paclitaxel resistant clones, we also identify potential modifiers of ABCB1-mediated resistance. Hence, this study establishes a robust, flexible and adaptable system for identifying drug resistance.

## Methods

### Plasmid construction

Transposon plasmid PB-SB-PGK-neo-bpA and transposase plasmid pCMV-PBase were obtained from Pentao Liu of the Wellcome Trust Sanger Institute. This plasmid was designed as an insertion mutagen that disrupted the structure of the inserted host gene. Several changes were made in PB-SB-PGK-neo-bpA to convert it to an activating mutagen. The plasmid is first digested with HindIII restriction enzyme and calf intestinal phosphatase, and ligated with a PCR-amplified fragment containing the CMV enhancer and promoter sequence [22] and the splice donor from the rabbit beta-globin intron [23] to make pPB-SB-CMV-neo-SD. The pPB-SB-CMV-neo-SD plasmid was then digested with BglII and XmaI to remove the PGK-Neo-bpA cassette, and was ligated with a PCR-amplified SV40-driven puromycin cassette to provide a rapid selection marker to identify successful integrants. The final plasmid was sequence-verified and named pPB-SB-CMV-puro-SD.

### Cell line transfection for library construction

To make a library, 1 × 10^7^ cells were plated overnight in four T175 flasks at cell density of 1 × 10^5^ cells per ml. HeLa and MCF7 were cultured in Dulbecco’s Modified Eagle Medium (DMEM) supplemented with glutaMAX (Invitrogen) and 10% fetal bovine serum (FBS). T47D was cultured in RPMI with glutaMAX and 10% FBS. IMR32 was cultured in Eagle’s Minimum Essential Medium (EMEM) supplemented with 10% FBS. Cells were co-transfected with 36 μg pPB-SB-CMV-puro-SD and 36 μg pCMV-PBase plasmids using 216 μl Fugene 6 (Roche) and 4.5 ml serum-free OPTI-MEM. After three days, cells were treated with fresh media with 2 μg/ml puromycin and cultured for additional 7–10 days. Cells surviving antibiotics treatment were harvested and cryopreserved as transposon-tagged prescreened libraries. In total, eight independent libraries were constructed, two for each cell line. To measure the insertion numbers per cell, cells from the original prescreened HeLa library were diluted and plated in a 96-well plate at average one cell per well. Five single cell colonies were identified, expanded and harvested for analysis.

### Transposition efficiency

To determine transposition efficiency, cells were transfected as above. One day after transfection, one cell plate was trypsinized and re-plated to a 6-well plate at various dilution ratios. Cells were treated with puromycin three days after transfection until colonies could be stained with Methylene Blue for visual counting. Transposition efficiency was defined as the proportion of initially seeded cells that could form puromycin-selected colonies.

### Paclitaxel screen

One million transposon-tagged cells from each library were plated in 100 mm tissue culture plates for drug treatment. Native untagged cells were similarly plated as study control. Paclitaxel dosages were 20 ng/ml for HeLa and MCF7, 15 ng/ml for T47D and 4 ng/ml for IMR32. Dosages were chosen as to sufficiently kill all parental cells within one week. Cells were treated until paclitaxel-resistant colonies were visible. Treatment time varied among cell lines depending on proliferation rates, and usually took ten days up to two weeks. Cells were then either harvested as resistant clones, or as resistant pools. To isolate resistant clones, colonies were picked from the drug-treated plates using 3 mm diameter cloning discs (Sigma), and expanded in 6-well plates in the presence of puromycin and paclitaxel. Cell clones exhibited stable resistance to both paclitaxel and puromycin, continuing to grow when retreated after 2 weeks culture in the absence of either agent. To harvest resistant pools, cells from the paclitaxel-treated plates were trypsinized and replated in the presence of puromycin and paclitaxel for one more week to remove any remaining non-resistant cells. These screens were performed on all eight libraries, including replicate screens for one library of each cell line.

### Splinkerette PCR and nextgen sequencing for insertion site detection

Genomic DNA was harvested from samples using DNeasy Blood & tissue Kit (Qiagen). Insertion sites can be detected by splinkerette PCR, a modified version of ligation-mediated PCR [24]. For the HeLa prescreened library, 3.3 μg genomic DNA was digested with 10 units of Csp6I (Fermentas) at 37°C for two hours, and ligated to 100 picomole double-stranded linker catalyzed by 2000 units of T4 DNA ligase at 16°C for overnight. The ligated sample was amplified with primers LP1 and PB51-IL in a 100 μl PCR reaction. Primer LP1 matches the linker sequences, and primer PB51-IL matches the transposon sequences. The thermo-cycling condition is the following: 3 min/94°C, 10 cycles of 15 sec/94°C; 30 sec/72°C with −1°C touchdown/cycle; 1 min/72°C, 20 cycles of 15 sec/94°C; 30 sec/62°C; 1 min/72°C, and 20 min/72°C. One microliter of the first PCR product was re-amplified in a 50 μl PCR using nested primers LP2a and PB52-ILa that contain Illumina single-end reaction adapter sequences for binding to the flowcell. Thermo-cycling condition was similar to that of the first PCR with 10 touchdown cycles and 10 regular cycles. Amplified products were purified using QIAquick PCR Purification Kit (Qiagen). For paclitaxel resistant pools and clones, 170ng genomic DNA was digested with 2 units of Csp6I and ligated to 10 picomole linkers. Up to 96 samples were processed with barcode linkers in a multi-well plate. Samples were pooled after PCR and purified. Sequencing was performed using Illumina HiSeq 50 Single Read following standard protocols except that sample loading density was reduced by 50% to avoid over-clustering due to the first 10 repetitive nucleotides. Each multiplexed cohort is loaded in one lane of the flow cell. Custom sequencing primer Seq-P1 matches the linker sequences prior to the barcodes and the read direction is opposite to CMV (Additional file 1: Table S1). Sequencing data were de-multiplexed and trimmed to remove the barcode plus 1 adjacent base remaining from ligation at the Csp6I half site, and any library adapter sequence present at the 3’ end of each read was removed. Reads of 7 bp or longer were retained and aligned to the hg19 reference genome using Bowtie alignment program [25], keeping only unique alignments placing the 5’ end of a trimmed read within 3 bp of a Csp6I site. All further analysis performed on the read counts at each Csp6I site.

### TOPO cloning and sanger sequencing

Nested PCR products of resistant clones (7 from MCF7, 1 from HeLa, and 4 from T47D) were prepared as above and cloned into vector pCR2.1-TOPO (Invitrogen). Bacterial colonies were sequenced with primer PB5-ILseq (Additional file 1: Table S1) from the transposon side. Insertion sites were aligned using the BLAT function of the UCSC Genome Browser version hg19 (http://genome.ucsc.edu/cgi-bin/hgGateway).

### Quantitation of mRNA expression

Total RNA was isolated using Qiagen RNeasy Mini kit. One microgram of total RNA was treated with RNase-free DNase to remove genomic DNA. First-strand cDNA was synthesized using Roche Transcriptor First Strand cDNA Synthesis kit, and quantitated by BIO-RAD SYBR Green. All reactions were normalized to actin.

### Detection of chimeric mRNA

To detect the chimeric mRNA, mRNA was reverse-transcribed as above. 1 μl cDNA was PCR-amplified using a forward primer specific to the PB transposon sequence and the reverse primer matching the ABCB1 exon 3 sequence. The thermo-cycling conditions are: 3min/94°C, 30 cycles of 30 sec/94°C; 30 sec/55°C; 30 sec/72°C, and 5 min/72°C. PCR products were fractionated on 1.7% agarose gel. The control PCR used the primer pair provided in the cDNA synthesis kit to amplify the housekeeping gene hPBGD for 35 cycles with 50°C annealing temperature, and the PCR products were fractionated on a 3% gel.

### Paclitaxel sensitivity assays

IMR32 Cells were reverse-transfected in a 96-well plate with either a control pCMV plasmid or pCMV6-ABCB1 plasmid (Origene). Each well contained 100 ng plasmid DNA, 0.3 μl Fugene 6 transfection reagent, and 10 μl OPTI-MEM, and 10,000 IMR32 cells in 100 μl antibiotics-free complete EMEM were seeded to each well. After two days, medium was replenished and cells were treated with serial-diluted paclitaxel for five days. Each sample was assayed with four replicate wells. Viability was measured by CellTiter-Glo (Promega) and data were processed using GraphPad Prism. Error bars represented standard error of means (SEM, n=4).

### Western blot

IMR32 cells were transfected with a control pCMV plasmid or pCMV6-ABCB1 plasmid respectively. Transfection was performed in a 6-well plate with each well containing 2 μg plasmid DNA, 6 μl Fugene 6 transfection reagent, 100 μl OPTI-MEM, and 200,000 IMR32 cells in 2 ml EMEM. After three days, cell were lysed with NP40 cell lysis buffer (Invitrogen) and sonicated to shear genomic DNA. Samples were diluted in SDS sample loading buffer, fractionated by SDS-PAGE (Bio-Rad), and transferred to polyvinylidene difluoride membrane. The membrane was blotted with MDR1/ABCB1 rabbit polyclonal antibody (Cell Signaling Technology #12273) diluted by 2,500-fold, and goat anti-rabbit IgG (Thermo Scientific #31460) diluted by 10,000-fold. For actin controls, the membrane was blotted with anti-actin rabbit monoclonal antibody diluted by 2,500-fold (Cell Signaling Technology #4970) and goat anti-rabbit IgG by 10,000-fold. Images were captured by G:Box (Syngene).

### Statistical and bioinformatics methods

To identify potential insertion sites in analysis of resistant pools and clones, we first filtered sequencing data to exclude ‘background’ signal derived from contaminating non-resistant cells or the low incidence of PCR products from inappropriate linker reactions or PCR reactions. We assumed that such background signal would follow a Poisson distribution. This was supported by our observation that a frequency distribution of sequence analysis from resistant cells followed a bi-phasic distribution, with a large number of different sequences represented at low frequency (1–50 reads) which resembled a Poisson distribution, combined with a series distinct sequences present at high frequency (100 reads upwards). We selected sequences present at >100 reads for further analysis, which we estimate represents significant enrichment (*p* < 0.05) over background signal. For analysis of pools of resistant cells insertion sites and targeted genes were then compiled between all samples, removing any insertions seen twice in repeated analysis of the same sample. For analysis of sequences from resistant clones, samples were further filtered to identify the 1–10 sequences present at highest frequency in each clone, based on our previous analysis of the likely number of transposon insertions per cell. Clones were then clustered manually based on shared insertion sites, and any samples clearly derived from more than one clone excluded. Data were then visualized using Gene Pattern software (Broad Institute of MIT and Harvard).

We use the Database for Annotation, Visualization and Integrated Discovery (DAVID) tool to perform functional analysis on genes enriched in the resistant pools (http://david.abcc.ncifcrf.gov). Only candidate genes identified above were used for analysis. Enriched genes were both listed as clusters and as an annotation chart.

To estimate the number of insertions needed to cover the genome, we assumed that only forward strand insertions within 64kb upstream could activate a gene based on our observation. We further postulated that the random event of integration within this 64kb region followed Poisson distribution. To achieve at least 1 insertion in 95% of total genes, the expected mean occurrence needed to be 3.0 [P (3.0, ≤0) = 0.05], which translated to 21.3kb gap between two insertions. Assuming genome size of 3 × 10^6^ kb, 1× genome coverage would need 2.8 × 10^5^ insertions. To achieve 2× coverage, the expected mean would be 4.75 [P (4.75, ≤1) = 0.05], equivalent to 4.4× 10^5^ insertions.

## Results

### Construction of gene activating transposons and generation of libraries of mutant cells

**Figure 1:**
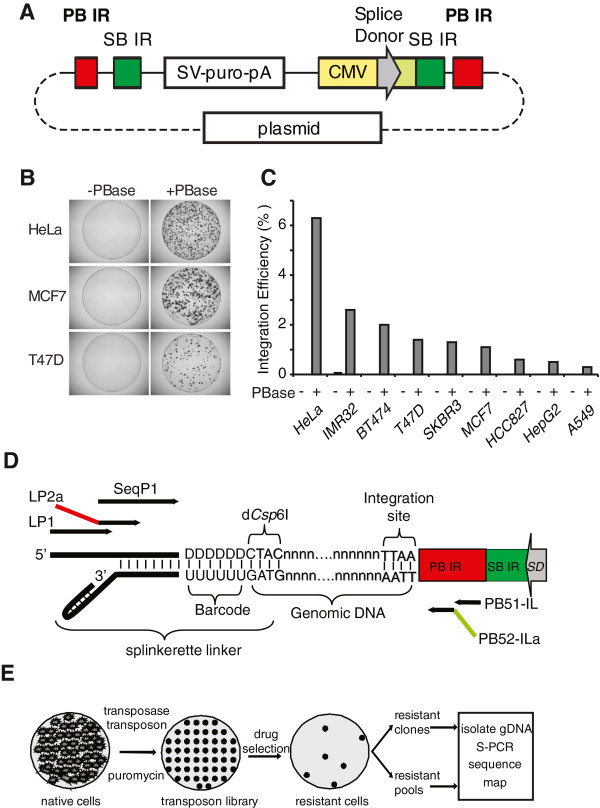
**Transposon mutagenesis libraries for forward genetic screens. A**) Diagram of PB plasmid pPB-SB-CMV-puro-SD. Inverted repeats (IRs) for the PB and SB transposons are shown. The cytomegalovirus enhancer and promoter is drawn as CMV. The rabbit β-globin splice donor is depicted with an arrow indicating its reading outward into adjacent genes. The construct is in a pBluescript-based plasmid vector. **B**) Transposase is required for transposon integration. Cells were transfected with PB plasmid in presence (+PBase), or absence (−PBase) of transposase plasmid followed by puromycin treatment. **C**) Transposition efficiency. Shown are PB transposition efficiencies with and without transposase. **D**) Splinkerette PCR template for insertion site detection. Nested PCR primers contain Illumina adaptors shown as red and green. A 6nt region in the linker (DDDDDD) serves as multiplexing barcodes. **E**) Mutagenesis and screen flow chart. The mutagenesis prescreened library was generated by transfection and expanded. Following drug selection, resistant samples were either isolated or pooled, and the insertion sites were identified by splinkerette PCR, Illumina sequencing, and mapping to a model genome.

Classic transposons consist of two functional components: a pair of short terminal repeats that target the host genome, and a transcribed transposase enzyme that catalyzes integration/ excision. Packaging these two elements separately allows experimental manipulation of transposons. The dual function transposon plasmid PB-SB-PGK-neo-bpA [26] that we obtained contains piggyBac/ Sleeping Beauty (PB/SB) terminal repeats for both transposons. This plasmid also contains a PGK promoter-driven neomycin selection marker for selection but lacks other transcription elements to activate host genes. Integration of this plasmid therefore can only disrupt the structure and expression of the host gene. To convert this plasmid to an activator mutagen, we added the cytomegalovirus (CMV) enhancer and promoter sequence, and a splice donor sequence, between the PB/SB inverted repeats (Figure 1A). The CMV enhancer and promoter contained a canonical TATA box and a strong upstream activation sequence that together can initiate strong transcription. The splice donor is able to combine with host splice acceptor downstream of the insertion site to generate a functional chimeric RNA. This generated a new transposon designed to have long range activation effects on gene expression when inserted in the forward orientation 5’ of the first coding exon. Furthermore, the transposon may also cause less predictable and short-range effects when inserted in the reverse direction or intragenically. Although the SB repeats were left intact, we only chose to use the PB to generate mutated libraries in a range of human cell lines, due to its higher efficiency and lower insertion site bias compared with SB [13,26]. Cells were co-transfected with PB transposon and transposase plasmids, and selected for puromycin resistance (Figure 1B). When co-transfected with transposase, transposons were stably integrated into cells at a frequency of between 6.3 and 0.3% of the starting population of cells (Figure 1C), whereas no integration was seen when transposons were transfected alone. The transposition frequency observed in HeLa cells was similar to that published by others [13] and the lower frequency we saw in other cell lines most likely reflects the relative efficiency of transfection with the plasmids. We selected 4 cell lines, HeLa cervical cancer cells, IMR32 neuroblastoma cells, MCF7 breast cancer cells and T47D breast cancer cells, for generation of libraries. For each cell line we transfected 10^7^ cells, generating libraries of 1–6 × 10^5^ independent elements. The insertion sites could be detected by splinkerette PCR and Illumina next generation sequencing (Figure 1D). We then went on to generate transposon mutagenized libraries; screen with a selection reagent; detect the insertion sites in resistant samples; and finally link the insertion events (genotype) to the resistance (phenotype) (Figure 1E).

### Characterization of insertion libraries

**Figure 2:**
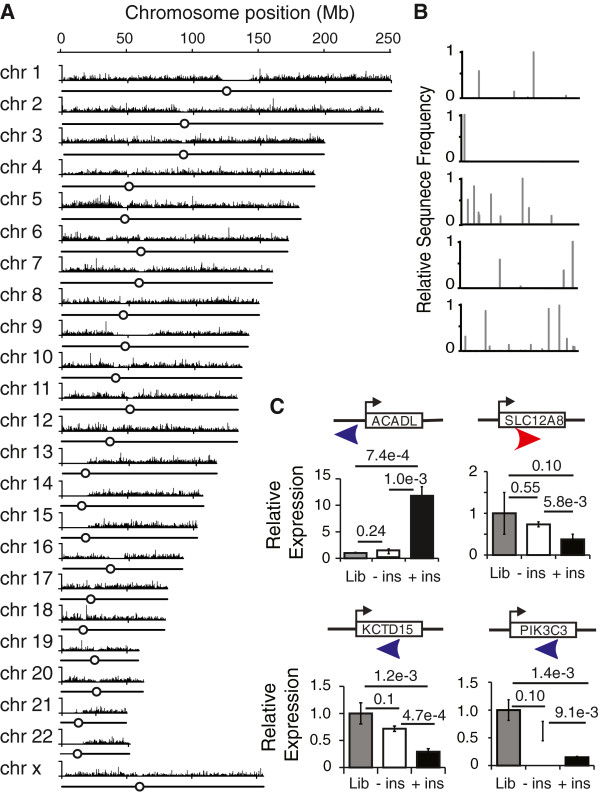
**Transposon Mutagenesis library. A**) All PB insertion sites in a PB-tagged HeLa library identified by Illumina sequencing were plotted to 23 chromosomes. X-axis indicates nucleotide positions with centromeres drawn as circles and chromosome arms as straight lines. Y-axis indicates raw read number for each site. **B**) Insertion sites of five clones expanded from single cells, with x-axes indicating genome positions, and y-axis indicating frequency of insertions normalized to the highest signal **C**) Transposon insertions alter host gene expression. Shown are four genes with PB insertions in various positions and orientations. Gene expression was compared among clones with (+ins), without (−ins) the insertion, and the prescreened library (Lib). Error bars show standard deviation (n=3). Significances were indicated by p-values.

To determine the extent of genomic distribution in our PB transposon libraries and provide a reference to subsequent chemotherapy resistant samples, insertion sites from a HeLa cell library were analyzed using Illumina next generation sequencing (Figure 2A). 4.6 × 10^5^ unique insertion sites were identified corresponding to 2.4% of all 19,228,691 TTAA integration sequences in the hg19 human genome. Insertion sites were characterized for their distribution throughout the genome and proximity to genes (Additional file 2: Dataset S1). This indicated widespread coverage of insertions throughout the genome, without any clear ‘hotspots’. Mean distance between insertion sites was 6.7kb, and 99.5% of gaps between insertions were of less than 44.5 kb. Few insertions were seen in the structural DNA of centromeres, or in the short arms of some chromosomes. This is expected due to the presence of heterochromatin and highly repetitive sequences that reduce insertions and confound analysis of any insertions that could occur. As very few annotated genes are located in these regions, the impact of the effective lack of insertions in these regions on functional mutagenesis is likely to be minimal.

Although a previous smaller study reported a preference of PB for transcribed genes with 70 out of 104 insertions being intragenic [13], our study found that just 45.6% of total insertion sites were located within transcribed gene sequences. This observation was consistent with the fact that 40.8% of all TTAA sequences in the genome are intragenic, indicating that there was no major preference for the transposon to insert into transcribed sequences. In addition, particularly relevant for our gene activation strategy, given that our data (described below) indicate that the transposon can, at least in some instances, activate expression of genes at a range of up to 64kb, we found 63% of insertions were within 25kb of at least one gene, an arbitrary range we chose to assign genes to insertion sites. Furthermore, we found that the proportions of sense- versus antisense- strand insertions are equivalent, both for the 63% insertions and for all insertions, indicating that transcribed sequences did not affect insert orientations.

To gain comprehensive identification of insertions within individual cells, 5 clones from the Hela prescreened library were isolated and sequenced, using DNA barcoding (Figure 1D and Additional file 1: Table S1) to permit multiplexing of samples. We found that each colony contained between 1 and 11 insertions, with an average of 6 insertions (Figure 2B). Based on this result, we estimate that there may be up to 3.8 × 10^6^ genomic insertion sites in our HeLa library of 6 × 10^5^ independent clones. However, only a fraction of these insertions were revealed by our Illumina sequencing of the library, likely due to technical limitations of the amount of genomic DNA used as input or the efficiency of the PCR reactions.

The generation of cell clones also provided the opportunity to explore the effects of transposon insertion on gene expression, which is the key to our functional mutagenesis approach As illustrated by our analysis of ABCB1 in the next section, ‘sense’ insertions upstream of genes consistently resulted in increased expression as expected. In one clone in which the transposon inserted upstream of the gene in the reverse orientation, expression was also increased (Figure 2C ACADL). In contrast, intragenic insertion of the transposon caused decreased expression. Based on this characterization of individually targeted genes, we conclude that our ‘activation tagging’ approach will result in consistent strong stimulation of gene expression when inserted in the forward orientation upstream of genes, coupled with less predictable repression of expression for reverse direction and/or intragenic insertions.

### Use of paclitaxel resistance screen to demonstrate the transposon functional mutagenesis approach

**Figure 3:**
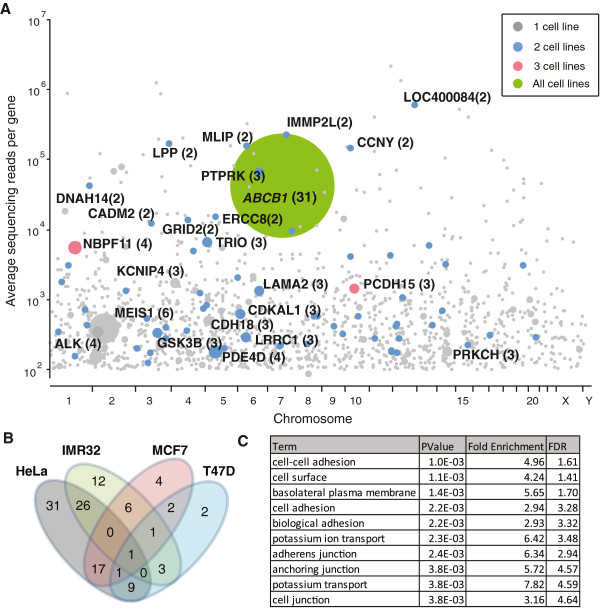
**Paclitaxel resistant gene candidates in pooled samples. A**) Candidate ‘hits’ identified in resistant pools of four cell lines. Genes found in multiple cell lines are color-coded and labeled. Dot surfaces and numbers within parentheses indicate insertion occurrence, and y-axis indicates total read numbers for each gene. **B**) Venn diagram showing candidate genes belonging to four cell lines. Only one gene (ABCB1) was shared by all four cell lines. **C**) Functional annotation analysis of pooled samples. Only genes with multiple hits were used in DAVID analysis. Only annotation groups with significant values (p-value<0.05, FDR<5%) are listed. The complete annotation chart and cluster chart are presented in the Additional file 4 Dataset S4.

Paclitaxel (taxol) is a well-defined microtubule interfering reagent broadly used in current chemotherapeutic regimens. Mechanism of resistance to paclitaxel includes elevated efflux pumps that reduce intracellular drug accumulation. The four transposon mutagenized cell libraries described above were treated with concentrations of paclitaxel sufficient to kill all the parental cells, and in all cases, paclitaxel-resistant clones emerged. Although drug-induced resistance could occur in native cell lines, we chose to initiate the screen with high dosages of drug, and screened for a relatively short period of time to prevent this effect. We found almost no surviving cells from native cell lines screened in parallel. In transposon treated cells being screened, colonies were usually identifiable as early as the background sensitive cells were cleared, indicating these resistant colonies were derived from genetically stable clones in the transposon mutagenized libraries. Transposon insertion sites in pools of resistant cells from each screen were then identified by Illumina sequencing and linked with nearby genes or other genomic features such as miRNAs (Additional file 3: Dataset S2). Sequencing data were first filtered to identify reads significantly (*p*<0.05) enriched over background signal using a Poisson-based test. To assess reproducibility, the screen was repeated with the original libraries, and again with independently generated transposon-tagged libraries. Combined data from these screens are presented in Figure 3A. Across all screens in the four cell lines, we identified 1,654 distinct insertion sites that were significantly enriched over predicted background signal, suggesting they are genuine transposon insertion sites found in the resistant cells. Of these 1,060 could be mapped to 916 different known genes or transcripts. Most genes were associated with a single transposon insertion across all the screens and probably represent ‘passenger’ mutations that are present in resistant cells but do not contribute to resistance. Only 115 genes were associated with multiple transposons, with 16 associated with 3 or more independent insertions. We also saw considerable agreement in genes between different cell lines, with around half of the genes identified in IMR32, MCF7 or T47D cells also identified in HeLa cells (Figure 3B).

### Identification of *ABCB1* as the benchmark resistant gene in all cell lines validated the mutagenesis screen

**Figure 4:**
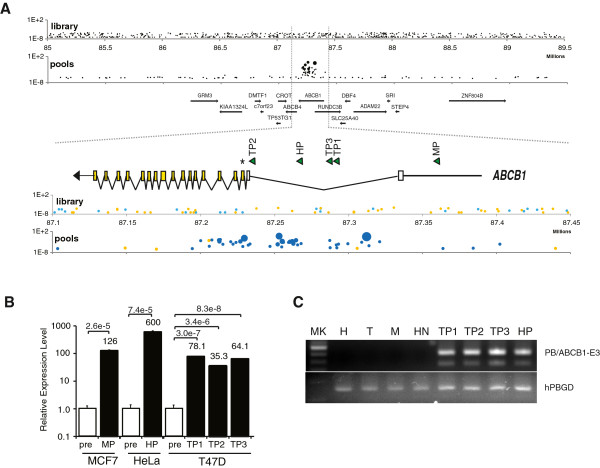
**ABCB1 as the primary resistant gene. A**) Insertion sites near *ABCB1* genomic locus are enriched in resistant samples. Insertion sites of a prescreened library and resistant pools in Chr7:85000000–89500000 are plotted. Scale is drawn as per Mb. Dot surfaces indicate number of positive samples, and y-axis indicates normalized read numbers as a percentage of total signals. Read numbers are unfiltered. All annotated genes within this region are shown as arrows. A blow-up view indicates *ABCB1* genomic arrangement with open reading frame shown as yellow boxes. Asterisk denotes exon 3 with the ATG start codon (chr7:87229506). Insertion sites confirmed by TOPO cloning and Sanger sequencing are drawn as triangles above the diagram with direction of arrows indicating orientation of the CMV and the splice donor. HP, MP, TP1-3 denote HeLa, MCF7, and T47D paclitaxel resistant clones respectively. Colors of dots indicate orientation of the CMV with forward orientation relative to *ABCB1* as blue and reverse orientation as yellow. **B**) PB insertions activate *ABCB1* expression. Error bars show standard deviation (n=3). Significances are indicated by p-values. “pre” denotes prescreened libraries; MP, HP, and TP1-3 denote clones shown above. **C**) Detection of the chimeric mRNA in clones with insertions in the ABCB1 intron. Top panel (PB/ABCB1-E3) shows 404bp chimeric PCR products with a transposon-specific primer and an ABCB1 exon 3 primer. A lower band at 300bp could be due to alternative splicing. Bottom panel (hPBGD) shows the 151bp PCR products using the primer pair amplifying the porphobilinogen deaminase (PBGD) housekeeping gene. Three native cell lines (H, HeLa; M, MCF7; T, T47D) and a HeLa clone with PB insertions but not in the ABCB1 gene (HN) were used as controls. The first lane (MK) indicates 100bp DNA ladder (New England Biolabs).

Multidrug resistant gene MDR1/ABCB1 is a well-known major contributor of resistance [27]. *ABCB1* was the only gene associated with multiple insertions (31 independent insertions at 25 different TTAA sites) in all cell lines tested. This enrichment was not seen in the parental libraries, but only after paclitaxel selection, clearly indicating ABCB1 as a causal factor for paclitaxel resistance (Figure 4A). Furthermore, after selection, insertions were clustered upstream of the gene open reading frame and most were oriented with CMV promoter and splice donor in the same direction as the *ABCB1* gene, as previously predicted to result in increased expression. To confirm that this was the case, we identified individual clones with insertions in *ABCB1* from 3 different cell lines and confirmed transposon insertions in 5 clones (1 in HeLa, 3 in T47D, 1 in MCF7) both by Illumina sequencing and by Sanger sequencing*.* For all of the insertion sites tested, transposon insertion led to increased expression of *ABCB1* mRNA (by 35- to 600-fold) over the prescreened library as determined by qPCR (Figure 4B). Of note, this included 1 clone (TP1) in which the transposon was inserted 64kb upstream of the open reading frame, indicating that ‘activation tagging’ can work at considerable distances and does not appear to involve the endogenous gene promoter. The significant enrichment for insertions at genomic position near *ABCB1* predicted (and for some confirmed) to lead to increased expression therefore provides strong validation of this screening method for finding relevant resistance mechanisms. We further detected the presence of chimeric mRNA which contained both the transposon and ABCB1 gene sequences in clones with insertions in the ABCB1 intron, but not in native cells (Figure 4C).

While several other genes were associated with multiple transposon insertions (Figure 3A), these genes were represented at significantly lower levels than *ABCB1* (with none having greater than 6 independent insertions) and none were seen in all cell lines tested. To test whether the screen approach can enrich cell processes and pathways related to paclitaxel resistance, the 115 genes with ≥2 insertions were analyzed by functional and structural motifs using Database for Annotation, Visualization and Integrated Discovery tool (DAVID) (Additional file 4: Dataset S4) [28]. There was strong enrichment for genes associated with microtubule components and cytoskeletal rearrangement, which are known paclitaxel targets (Figure 3C) [29-31]. Ion transport channels were likewise enriched, consistent with reports that ion channels utilize microfilaments for their function and are susceptible to paclitaxel, and that their expression can affect sensitivity to paclitaxel [32-38]. Thus, taken together, these results show that our transposon mutagenesis approach can readily identify major resistance mechanisms and provide potential insight into the biological processes targeted by the drug used to select resistant cells.

### Use of clonal analysis to reveal gene interactions

**Figure 5:**
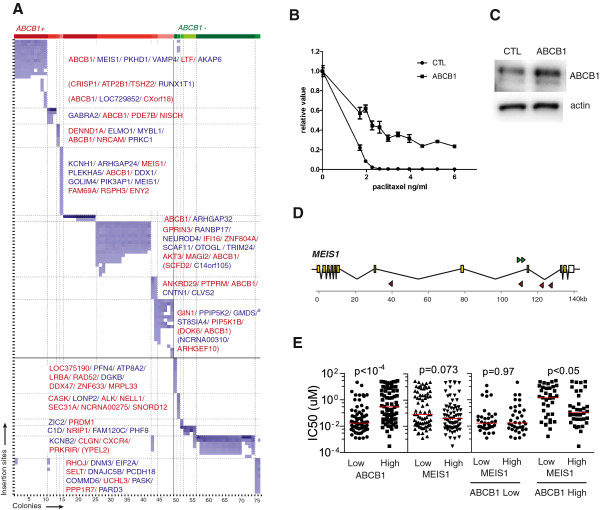
**Candidate hits in resistant clones. A**) Cluster analysis of IMR32 resistant clones. X-axis indicates colonies and y-axis indicates insertion sites. Colonies within a cluster have same insertions and are likely derived from one founder clone. Insertions are in either same (red) or opposite (blue) orientations of a gene. **B**) Paclitaxel sensitivity curve of IMR32 transfected with a control (CTL) or *ABCB1* cDNA plasmid (ABCB1). Cell survival was measured by CellTiter-Glo assay. **C**) Western blot showing ABCB1 overexpression in IMR32 cells transfected with pCMV-ABCB1 plasmid. CTL, cells transfected with a control pCMV plasmid; ABCB1, cells transfected with pCMV-ABCB1 cDNA plasmid. ABCB1 was shown as bands at 150kD. Actin was used as loading control. **D**) PB insertions in *MEIS1* gene. The direction of gene is drawn from left to right, with yellow squares indicating exons. Forward strand insertion sites are drawn as green triangles and reverse as red triangles. Scale is drawn as per kb. **E**) Paclitaxel sensitivity profile in a panel of cancer cell lines. Cell lines are either divided to two groups by median *ABCB1* or *MEIS1* mRNA levels, n=143, (first and second graphs), or first divided by median ABCB1 levels and then by median MEIS1 mRNA levels, n=72 (ABCB1 Low) and 71 (ABCB1 High). Red bars indicate median IC50.

To complement the analysis on resistant pools, we also isolated and sequenced cell colonies from a resistant pool of IMR32 cells (Additional file 5: Dataset S3) to gain a deeper understanding of the insertions that may drive paclitaxel resistance for individual clones. 82 clones were isolated and sequenced. After sequence analysis, 7 were found to be derived from more than one originating clone and were excluded from further analysis. The remaining 75 were used for clustering analysis. Clustering analysis revealed that these clones appeared to be derived from at least 14 distinct originating clones (Figure 5A). 8 of these originating clones carried insertions in *ABCB1* (*ABCB1+*), including clones containing only *ABCB1* insertions, suggesting that it alone is able to drive resistance in the context of this cell line. To confirm this, we overexpressed *ABCB1* in IMR32 cells by cDNA plasmid transfection and demonstrated increased resistance associated with *ABCB1* overexpression (Figure 5B, C).

Furthermore, the second most common hit in our pool analysis (Figure 3A), a transcription factor *MEIS1*, was only selected in IMR32 cells, and in clonal analysis was only seen in clones that also had insertions in ABCB1, implicating a possible role for *MEIS1* in modifying *ABCB1*-mediated resistance, rather than inducing resistance alone. Insertion site orientation and positions of transposons suggested that enhanced resistance is associated with down regulation of *MEIS1* expression (Figure 5D), although we were not able to directly verify this using siRNA-mediated gene knockdown (data not shown). Therefore, to look for independent evidence of *MEIS1* function within *ABCB1* context, we turned to our recently published database of drug sensitivity for a panel of cancer cell lines [39] and the publicly available Broad Institute Cancer Cell Line Encyclopedia (CCLE) microarray database [40]. Our drug sensitivity database consists of 639 human cancer cell lines in combination with 130 targeted therapy or cytotoxic drugs, assayed in a 9-point 256-fold serial dilution setting. In total, 143 cell lines across diverse cancer types that have been assayed for paclitaxel sensitivity in our database overlapped with the CCLE cell line collection for gene expression, and were analyzed for correlation between paclitaxel sensitivity and expression of *ABCB1* and *MEIS1*. As expected, there was a significant correlation between *ABCB1* expression and paclitaxel sensitivity, but not between *MEIS1* and paclitaxel sensitivity (Figure 5E). Instead, a negative correlation was observed between *MEIS1* expression and paclitaxel sensitivity only in cell lines expressing high levels of *ABCB1*, but not in *ABCB1*-low cells, as predicted by clonal analysis. Although independent validation will be required to confirm the role of MEIS1 these data suggest that transposon activation mutagenesis and clonal analysis can be used to reveal interesting information such as primary resistant events and modifiers.

## Discussion

Acquired resistance to chemotherapeutic drugs remains a major hurdle to effective cancer treatment and eradication, and a better understanding of the genes and pathways that contribute to this is needed. Our data demonstrate that transposon mutagenesis provides a powerful, adaptable and cost-effective forward genetic approach for identifying resistance genes. The use of parallel screening in four separate cell lines with replicate samples to identify both common and cell-line restricted resistance gene candidates illustrates the potential of this system for gaining a deeper and more comprehensive view of resistance across the full spectrum of malignancy.

Transposon-based gene activation systems have a unique combination of properties that make them ideal for studies of tumor cell resistance. First, they are readily applied to gain-of-function genetic screens, unlike the vast majority of functional genetic interrogation approaches for mammalian genomes. This may be of particular relevance in tumors, where gene activation or amplification are common transforming events. The potential for long-range transcriptional activation and the need to hit only one allele of a gene in diploid cells provides higher effective genome coverage than gene inactivation strategies at comparable levels of mutagenesis. Furthermore, the presence of multiple effective mutations in single cells should allow for identification of ‘cooperating’ resistance genes, as suggested by our analysis of the *ABCB1/MEIS1* interaction in resistant clones. A second advantage is the potential to identify resistance events occurring from changes in expression of uncharacterized or poorly understood genetic elements such as long intergenic non-coding (LINC) RNA or microRNAs. We have identified resistant cells bearing insertions in LINC-RNAs and unannotated transcripts and further investigation of these mutations could shed light on new genetic elements. Third, this system is readily transferable to new cell lines, including cells derived from patient samples, and libraries can be expanded and regenerated simply by re-transfection with the transposase. Hence transposon-based screens allow for the rapid generation of resistant clones to single drugs or combined therapies in specific tumor cells, providing insights into potential resistance mechanisms. These could then be used to guide design of new drug combinations and tailor treatments to particular tumor types.

Transposon-based screening has been used previously to identify potential mechanisms of resistance to the antibiotic puromycin and chemotherapeutic, vincristine [41]. In those studies, transposon insertions were found primarily in *Abcb1a/b* (both drugs) and the closely related transporter *Abcg2* (puromycin only), reinforcing our findings that overexpression of *ABCB1* represents a major mechanism of drug resistance. However, additional candidate genes were not identified; this may be due to the absence of a splice donor in the transposon used, limiting the ability of the inserted promoter to activate gene expression, the presence of only one transposon per cell, or the relatively limited analysis of insertion events using capillary sequencing of isolated cell clones

Based on our finding that the transposon used here can exert a strong transcriptional activation effect at 64kb upstream from open reading frames, we estimate (using Poisson distribution) that libraries consisting of just 4.7 × 10^4^ clones (2.8 × 10^5^ insertions) or 7.3 × 10^4^ clones (4.4 × 10^5^ insertions) respectively could be potentially capable of activating 95% of genes by delivering at least one or multiple forward upstream insertions. Therefore in the case of HeLa cells we have approached meaningful close to genome-wide coverage for activation events. This is supported by our results with *ABCB1,* from which it is clear that all of our libraries had sufficient coverage to provide multiple insertions in a single strong resistance gene. We deduced that the low incidence of identification of other resistance genes could therefore reflect real differences in the ‘potency’ of individual genes to promote resistance, with only ABCB1 being sufficient while other events requiring additive effects of multiple genes to yield resistance in the high taxol concentration used here for selection. This is also supported by a prior transposon screen [41 described above] which identified only ABC-family transporters as potential resistance genes. Support for this also comes from our bioinformatics analysis, which revealed concordance of gene function or pathways between candidate resistance genes, with a strong enrichment in microtubule related biology previously linked to paclitaxel resistance.

However, even considering these potential limitations, our data identify new possible resistance genes and strengthen the evidence for previously identified candidates. As an example, clonal analysis of resistant cells strongly implicate *MEIS1* as a modifier of *ABCB1*-mediated resistance, and this is further supported by our analysis of a large panel of tumor cells. *MEIS1* is a class A homeodomain protein that acts as a cofactor for homeobox (HOX) proteins, and has been implicated as a critical downstream target of oncogenic fusion proteins in leukemia [42-45]. Although high expression promotes leukemia cell proliferation, silencing of *MEIS1* increases resistance to the chemotherapeutic etoposide [46], in agreement with our findings in IMR32 cells. Our clonal analysis of mutations also reveal *CXCR4*, the receptor for the chemokine CXCL12 (stromal derived growth factor 1, SDF-1), as a potential resistance gene that functions independently of *ABCB1* (Figure 5A). Up-regulation of CXCR4 is associated with increased metastasis and poor prognosis in various forms of cancer, in part due to effects on cellular phenotype, and is associated with chemotherapeutic resistance in numerous tumor models. For example, CXCR4 is upregulated in gefitinib-resistant non-small cell lung cancer cells and promotes epithelial-mesenchymal transition (EMT) and self-renewal activity [47]. Likewise, CD133^+^ glioblastoma cancer stem cells with increased resistance to a range of chemotherapeutic agents, including paclitaxel, express high levels of CXCR4 [48], and high surface expression of this chemokine receptor is considered a marker of cancer stem cells [49]. Of direct relevance to our results, increased CXCR4 expression and CXCL12/ CXCR4 signaling promote tumor cell resistance to the chemotherapeutic gemcitabine [50].

Other genes identified in our screen, such as *ALK* and *PDE4D*, increase tumor cell growth and protect from apoptosis, and may therefore promote resistance through these mechanisms.

Finally, further genes identified from independent hits in different cell lines, including the protocadherin *PCDH15* and neuroblastoma breakpoint family member NBPF11 have not been implicated in tumor resistance, but based on the examples described above, may also have important roles.

## Conclusions

We have developed a transposon-mediated activation mutagenesis and screen approach to systematically identify chemotherapy resistance. We demonstrated the feasibility of using this approach by identifying genes and pathways related to paclitaxel resistance. This system provided unbiased genome-wide coverage with sufficient depth to reliably capture the most well characterized mechanism of resistance in all cell lines processed and generate many believable additional hits based on available functional annotation. In addition to a dramatically lower cost and higher efficiency over RNAi and cDNA libraries, this approach does not require *a priori* knowledge of candidate genes, can survey untranscribed regions and can generate stable resistant clones pertinent to specific cell lines. Although further analysis will be required to dissect candidate genes, our findings highlight the potential for transposon-based functional genetics to aid in identifying both novel resistance genes and gene combinations. These may allow improved selection of chemotherapeutic drugs for particular classes of tumors, or the characterization of “resistance gene signatures” for new or ‘black box’ targeted therapeutics, allowing development of combination therapies to overcome potential resistance, and improve the efficacy and duration of new cancer therapies.

## Competing interests

The authors claim no financial competing interests or non-financial competing interests.

## Authors’ contributions

LC and EVS designed the research; LC performed the research. EVS, AL-H and LS provided guidance to the work; LC and AL-H analyzed data; MB and TKO performed sequencing and alignment; SB and GKV provided barcode linkers; AD and CB analyzed cell line IC50 and microarray panel; LC, AL-H, CB wrote the manuscript. AL-H and EVS were two senior authors contributing equally to this work. All authors read and approved the final manuscript.

## Pre-publication history

The pre-publication history for this paper can be accessed here:

http://www.biomedcentral.com/1471-2407/13/93/prepub

## Supplementary Material

Additional file 1: Table S1 Nucleotides used in the study. Listed are oligonucleotides used as ligation linkers and PCR primers. Li-EN U and Li-En D were annealed to generate barcoded splinkerette linkers with a sticky end compatible to the Csp6I-generated end. First round PCR primers were LP1 and PB51-IL, second round PCR primers were LP2a and PB52-ILa, sequencing primers were SeqP1 and PB5-ILseq. Barcode linkers were arranged as a 96-well plate. Click here for file

Additional file 2: Dataset S1 Insertion sites of a HeLa prescreened transposon library. Shown are all insertion sites identified by Illumina sequencing. Genes within +/-25kb range of the insertion sites are indicated plus orientation and positional information. Click here for file

Additional file 3: Dataset S2 Insertions in genes identified in selected pools of paclitaxel-resistant cells. The “AlignStats” table shows barcode assignment for each sample, as well as number of reads, number of aligned reads, and percentage of alignment. The “summed pools” table shows the number of independent insertions, the total number of sequencing reads and the average number of sequencing reads associated with a single gene in each screen. For each cell line, 3 independent screens were performed, using 2 libraries (labeled 1 and 2), with library 1 screened twice (1A and 1B) and library 2, once. To remove any bias arising from rescreening of the same library twice, any duplicate insertion sites identified in both screen 1A and 1B were removed, and data for 1A and 1B screens show only independent insertion sites. These data are represented as a bubble plot and Venn diagram in Figures 3A, B. Click here for file

Additional file 4: Dataset S4 Analysis of gene candidates by Database for Annotation, Visualization and Integrated Discovery (DAVID) tool. Genes identified by screen were fed to DAVID analysis tool (http://david.abcc.ncifcrf.gov/). Only candidate genes identified in at least two incidences in the Dataset S2 were used for analysis. Enriched genes were both listed as clusters and as an annotation chart. Click here for file

Additional file 5: Dataset S3 Insertions in isolated paclitaxel-resistant colonies from IMR32 cells. Table shows data for individual transposon insertion sites, expressed as (number of sequencing reads for insertion site/ total number of sequencing reads for colony) x 100. Data were filtered to remove any sites represented at < 1% of total reads for a colony. Data were then ordered based on transposon insertion sites. Also indicated are genes associated with each transposon insertion site, as well as the orientation of the insertion (‘sense’ indicates that the CMV promoter in the transposon is oriented in the same direction as the gene promoter) and position (upstream of first coding exon, within gene coding sequence or downstream of poly-adenylation site). These data are represented by the heat map in Figure 5A. Click here for file
