# Supplementary material for: Transposon activation mutagenesis as a screening tool for identifying resistance to cancer therapeutics
Source: BMC Cancer. 2013 Feb 27;13:93. doi: 10.1186/1471-2407-13-93 (PMC3598783; doi:10.1186/1471-2407-13-93)
Supplement: Additional file 1: Table S1 — Nucleotides used in the study. Listed are oligonucleotides used as ligation linkers and PCR primers. Li-EN U and Li-En D were annealed to generate barcoded splinkerette linkers with a sticky end compatible to the Csp6I-generated end. First round PCR primers were LP1 and PB51-IL, second round PCR primers were LP2a and PB52-ILa, sequencing primers were SeqP1 and PB5-ILseq. Barcode linkers were arranged as a 96-well plate. [file 1471-2407-13-93-S1.doc]

## List of oligonucleotides used

| Oligo | Sequences | Note |
| --- | --- | --- |
| Li-En D | GTAATACGACTCACTATAGGGCACGCGTGGTCGACTGCGCATDDDDDDC | Splinkerette down strand, DDDDDD for barcode down, in 96well format |
| Li-En U | TAGUUUUUUATGCGCAGTTTTTTTGCAAAAA | Splinkerette up strand, UUUUUU for barcode up, complementary to DDDDDD |
| LP1 | GTAATACGACTCACTATAGGGCACG | First round PCR primer matching Li-En D 5' half |
| PB51-IL | CGCTATTTAGAAAGAGAGAGCAATATTTCA | First round PCR primer matching PB LTR |
| LP2a | AATGATACGGCGACCACCGACACTCTactatagggcacgcgtggt | Second round PCR primer, UNDERLINED UPPERCASE matching flowcell adapter, lowercase matching Li-En D |
| PB52-ILa | CAAGCAGAAGACGGCATACGAGCTCTTCagaatgcatgcgtcaattttacgcagac | Second round PCR primer, UNDERLINED UPPERCASE matching flowcell adapter, lowercase matching PB LTR |
| SeqP1 | ACTATAGGGCACGCGTGGTCGACTGCGCAT | Sequencing primer for NGS, matching Li-En D immediately before barcodes |
| PB5-ILseq | CATGCGTCAATTTTACGCAGACTATCTTTC | Sequencing primer for TOPO-Sanger sequencing, matching PB LTR |

DDDDDD down strand barcodes

| AAGTGT | ACATTG | ACTATG | ACTGAG | AGAGCT | AGGCGT | AGGCTG | AGTACT | AGTGAT | AGTGTA | ATAGTG | ATATAT | A |
| --- | --- | --- | --- | --- | --- | --- | --- | --- | --- | --- | --- | --- |
| ATATGC | ATCAGT | ATCATG | ATGATC | ATGGAT | ATGTTG | ATTAAG | ATTGAC | ATTTAA | CATAGT | CATGAT | CATGTA | B |
| CATTAG | CCAACT | CCAGTT | CCTGGC | CCTTTG | CTACGT | CTATGA | CTCACG | CTCTGT | CTGGGC | CTGTCT | CTTCAG | C |
| CTTTGC | GACGCG | GACTAT | GAGCCA | GATATG | GATGTT | GCCTGG | GCCTTT | GCGTAG | GCTCAT | GCTTCA | GGATAT | D |
| GGCATG | GGCGTA | GGCTGA | GGGCAT | GGGCTA | GGGGCT | GGTGGT | GTCTAC | GTCTCT | GTGATA | GTGGTG | GTGTAA | E |
| GTGTGT | GTTCCA | GTTGAT | GTTTTT | TACGTC | TAGAGT | TAGGCG | TAGTAC | TAGTGA | TATCAG | TATGCA | TCAGCT | F |
| TCATGA | TCCAGT | TCTACG | TCTCTG | TCTGTC | TGACCT | TGACTA | TGATGT | TGCCTT | TGCTCA | TGGATA | TGGGCA | G |
| TGGGGC | TGGTGG | TGTAAG | TGTCTC | TGTGTG | TGTTGA | TTCAGC | TTCCAG | TTGATG | TTGCCT | TTTGCC | TTTTTG | H |
| 1 | 2 | 3 | 4 | 5 | 6 | 7 | 8 | 9 | 10 | 11 | 12 |  |
